# Case Report: Neonatal onset intractable diarrhea and hypoproteinemia due to intestinal malrotation and chronic midgut volvulus

**DOI:** 10.3389/fped.2024.1494599

**Published:** 2025-01-14

**Authors:** Cuifang Zheng, Song Sun, Ying Gong, Shijian Miao, Kuiran Dong, Ying Huang

**Affiliations:** Children’s Hospital, Fudan University, Shanghai, China

**Keywords:** chronic diarrhea, intestinal malrotation, volvulus, congenital diarrhea and enteropathies, protein-losing enteropathy, malabsorption syndrome

## Abstract

Congenital diarrhea and enteropathies (CODEs) are a rare heterogeneous group of inherited disorders that typically present with severe chronic diarrhea during the first weeks of life. As a broad range of illnesses can present similarly in infants, establishing a definitive cause for CODEs is challenging. In this report, two infants were suspected to have CODE, with neonatal-onset chronic diarrhea and protein-losing enteropathy finally found to be due to intestinal malrotation and chronic midgut volvulus. Although the disease onset time was during the neonatal period, the typical findings of intestinal malrotation and volvulus were not present. Following the diagnostic approach for CODEs, both patients underwent extensive examinations without a definitive diagnosis. Intestinal malrotation was incidentally detected by an abdominal CT examination. After surgical correction of the underlying malrotation (Ladd's procedure), both infants had resolution of their diarrhea and hypoalbuminemia.

## Introduction

Congenital diarrhea and enteropathies (CODEs) are a rare heterogeneous group of disorders that cause early onset intractable diarrhea, characterized by chronic diarrhea, malabsorption, and failure to thrive in infants. As a broad range of illnesses can present similarly in infants, the evaluation of CODEs is a lengthy process and may not lead to a definitive diagnosis. Patients with CODEs often have high morbidity and prolonged hospitalization. Although the established classification and diagnostic approach for CODEs has helped clinicians in evaluating and managing these rare disorders (1), establishing a definitive diagnosis of CODEs remains challenging.

Intestinal malrotation is a developmental anomaly of the midgut in which normal fetal rotation of the intestines around the superior mesenteric artery and their fixation in the peritoneal cavity fail. Bilious vomiting and bloody stools are the two most common clinical presentations in neonates with malrotation. Obscure or non-specific symptoms are common in patients beyond infancy (2). Herein, we report two infants with intractable chronic diarrhea within their first week of life, complicated by severe hypoalbuminemia, malnutrition, and growth retardation. Acquired diarrhea, including allergic disorders, lactose intolerance, chronic infectious enteritis, and neonatal necrotizing enterocolitis, were excluded. Both patients were suspected to have CODE. Esophagastroduodenscopy, colonoscopy, histological evaluations, immunologic investigations, and family-based whole-exome sequencing (WES) were conducted but did not lead to a definitive diagnosis. Neither infant had bilious emesis or bloody stools. Intestinal malrotation was incidentally detected on abdominal contrast-enhanced computed tomography (CT). Chronic midgut volvulus was confirmed during the laparoscopic Ladd's procedure. After Ladd's procedure, the main symptoms of chronic diarrhea and hypoalbuminemia were relieved.

This report reminds pediatricians to be aware of the possibility of intestinal malrotation when patients are suspected to be or are diagnosed with obscure CODEs, even in the absence of the classical hallmarks of the disease.

## Case presentation

### Case 1

The patient was delivered at full term from a non-consanguineous family with a birth weight of 2,930 g. She had initial symptoms of diarrhea at the age of 3 days. The stool frequency was 8–10 times/day, with the characteristics of watery diarrhea with some mucus in the stool and occasionally with a few blood spots. Lactose intolerance and a cow milk protein allergy (CMPA) were suspected, and lactase supplementation was initiated. For the full-breast-fed infant, her mother started a strict allergen avoidance diet (cow's milk, soya, egg, and nuts), but with little effect. An extensively hydrolyzed protein-based formula (lactose-free and low osmotic pressure of osmotic pressure is 198 mOsm/kg) was started at the age of 3 months, with little improvement in her symptoms. An amino acid-based formula (lactose-free) was then initiated but with limited success. Esophagastroduodenscopy, colonoscopy, and abdominal CT were performed at a local hospital without obvious abnormalities.

In February 2022, at the age of 6.5 months, she was transferred to our hospital with severe chronic diarrhea, intractable hypoalbuminemia, and failure to thrive. On admission, her weight and height were 5.3 kg (<P3, weight for age *Z* score −2.84) and 63.0 cm (<P5, height for age *Z* score −1.50), respectively. Her serum total protein and albumin were 28.0 and 18.4 g/L, respectively. She also had elevated transaminase levels, hypogammaglobulinemia, and coagulation disorders (see Table 1). There were no significant abnormalities in blood sodium, potassium, and chloride levels. Detailed information on the laboratory tests is presented in Table 1. She was suspected to have CODE with protein-losing enteropathy (PLE). Following the diagnostic algorithm for watery diarrhea, a fasting trial for 48 h was initiated, with a significant improvement in diarrheal output, which was suggestive of diet-induced diarrhea. Esophagastroduodenscopy, colonoscopy, histological evaluation, immunological investigations, and family-based WES were conducted. Esophagastroduodenscopy and colonoscopy revealed no abnormalities. A histological evaluation did not reveal any signs of autoimmune enteropathy (AIE), microvillus inclusion disease (MVID), or congenital tufting enteropathy (CTE). No pathogenic variants were detected by WES.

**Table 1:** Clinical laboratory data on admission.

	Case 1	Case 2	Reference value
Age (month)	6.5	7.5	—
Total protein (TP, g/L)	29.8	29.2	55–75
Albumin (Alb, g/L)	18.28	18.62	39–54
Hemoglobin (Hb, g/L)	131	105	97–141
Lymphocyte count (×10^9^/L)	2.09	1.19	2.50–9.0
pH value	7.38	7.33	7.35–7.45
Serum sodium (Na^+^, mmol/L)	132	133	136–146
Serum potassium (K^+^, mmol/L)	3.5	2.8	3.5–5.3
Serum chlorine (Cl^−^, mmol/L)	110	112	98–106
Alanine transaminase (ALT, U/L)	135.52	106.11	8–71
Aspartate transaminase (AST, U/L)	145.86	92.94	21–80
Immunoglobulin G (IgG, g/L)	0.70	1.4	3.5–8.9
Immunoglobulin M (IgM, g/L)	0.41	0.26	0.33–1.25
Immunoglobulin A (IgA, g/L)	0.24	0.29	0.06–0.54
Activated partial thromboplastin time (APTT, s)	69.5	45.2	28–45
International normalized ratio (INR)	1.53	1.13	0.8–1.2
Prothrombin time (PT, s)	18.6	14.6	11–14.5
Prothrombin time activity (PTA, %)	51.0	79.0	80–120
Fibrinogen (FIB, g/L)	1.55	1.34	2–4

**Figure 1:**
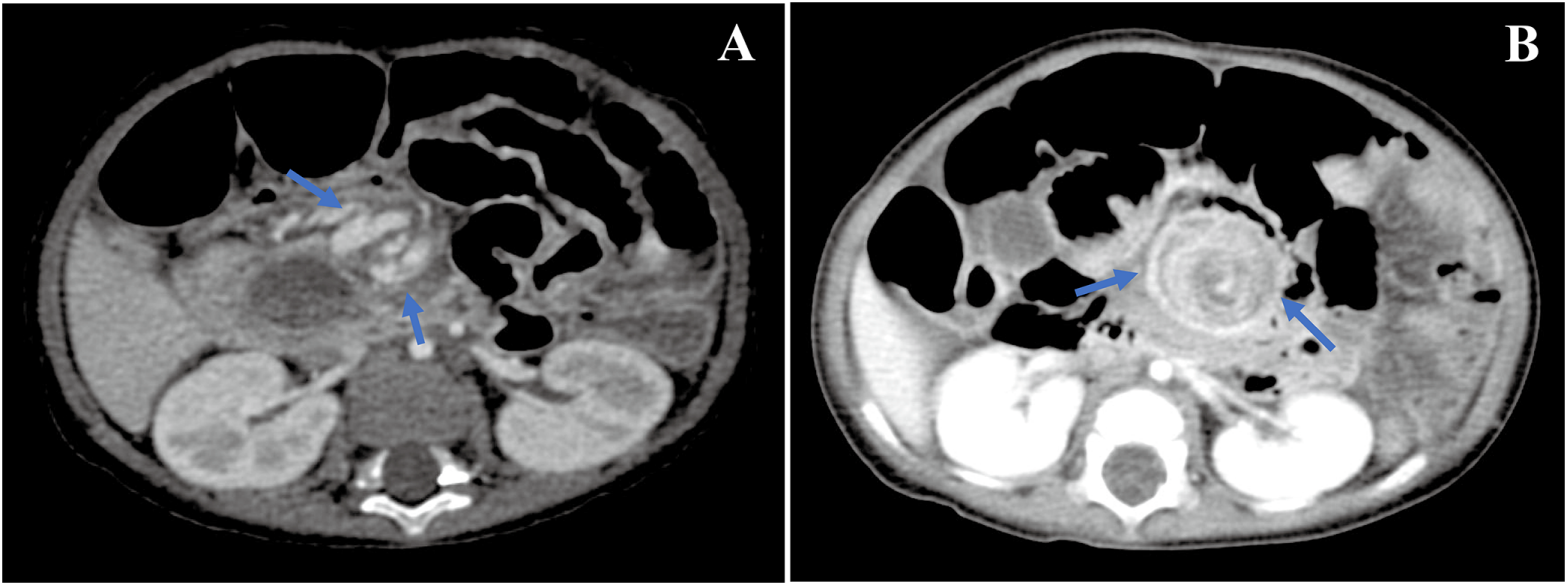
CT images. Whirlpool sign in the superior mesenteric vessel's branches was detected by an abdominal CT examination [**(A)** in case 1 and **(B)** in case 2].

Intestinal malrotation was incidentally detected on abdominal CT (Figure 1A). Upper gastrointestinal imaging (UGI) and transabdominal ultrasonography confirmed this finding. After an extensive discussion with our team members, we suspected that intestinal malrotation may be the cause of her symptoms, and she was referred to the Department of Pediatric Surgery for further treatment. During the laparoscopic procedure, intestinal malrotation with a 270° chronic midgut volvulus was detected. It was also found that a part of the small intestine had adhered to the sigmoid mesocolon. There were no signs of ischemia or necrosis in the small intestine. The patient underwent Ladd's procedure, adhesiolysis, and an appendectomy. Postoperatively, the main symptoms of chronic diarrhea and hypoalbuminemia were gradually relieved, and her weight gain improved. The diarrhea symptoms were completely relieved 4 months post-surgery. There was no need for a repeated infusion of albumin and immunoglobulin 1 year after the operation. The last follow-up was conducted 26 months post-operation. She had no symptoms of diarrhea. Without any additional medical treatment, the patient's serum albumin was normal, and her malnutrition gradually improved. Her weight and height were 13.5 kg (P25–P50, weight for age *Z* score 0.03) and 95.0 cm (P25–P50, height for age *Z* score 0.2), respectively.

### Case 2

The patient, who was born at full term and weighed 3,190 g, was the only offspring of healthy non-consanguineous parents. There were no medical conditions or reported teratogenic exposures during pregnancy. The patient had initial symptoms of diarrhea, fever, and non-bilious vomiting in his first week of life. Stool frequency was more than 10 times/day, characterized as watery diarrhea. Inpatient laboratory tests revealed hypoalbuminemia (see Table 1). A massive cystic mesenteric mass (5.9 cm × 5.1 cm × 6.3 cm) and intestinal malrotation were detected during a CT scan. An exploratory laparotomy was performed 15 days after birth at a local hospital. The cystic mesenteric mass was removed. An histopathological examination indicated mesenteric cystic lymphangioma. After surgery, the symptoms of diarrhea and hypoalbuminemia were not relieved. At the age of 4 months, he was presumed to have CMPA, and an amino acid-based formula (lactose-free) was initiated, but with limited effect. At the age of 5 months, esophagastroduodenscopy, colonoscopy, and histological evaluations were conducted at a local hospital, without obvious abnormalities. Physicians at the local hospital were unaware that intestinal malrotation may be the cause of his chronic symptoms.

**Figure 2:**
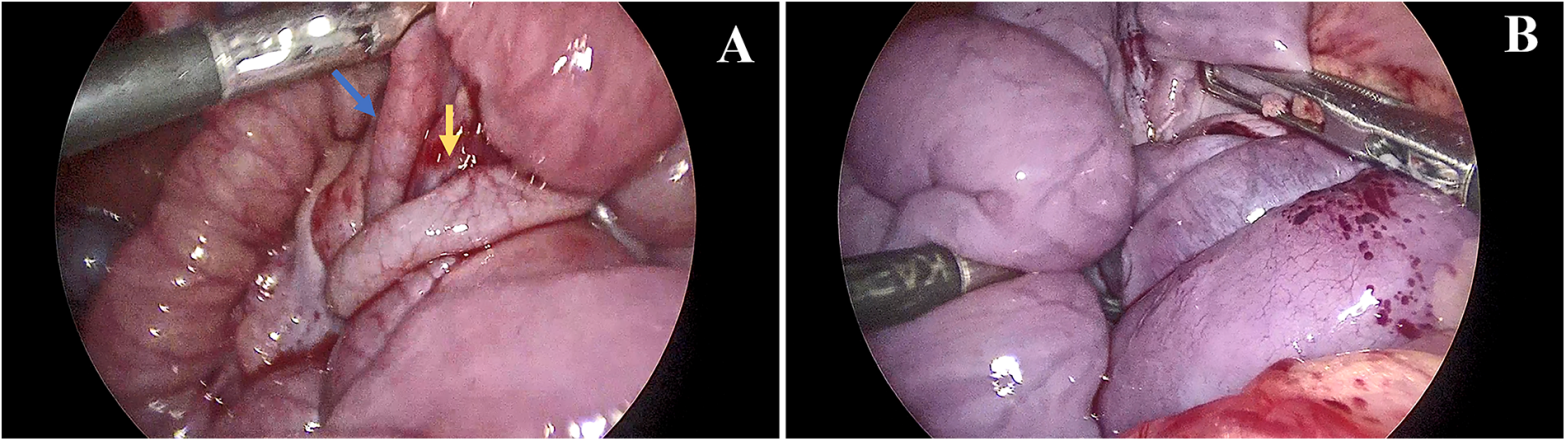
Laparoscopic views. The laparoscopic procedure revealed a 360° clockwise midgut volvulus in case 2. **(A)** An abnormally positioned appendix was noted in the upper abdomen (blue arrow), and the root of the mesentery was twisted as a whirlpool (yellow arrow). **(B)** The small bowel was noted to be ischemic without necrosis.

At the age of 7.5 months, he was transferred to our hospital with severe chronic diarrhea, hypoalbuminemia, and failure to thrive. On admission, his weight and height were 6.5 kg (<P3, weight for age *Z* score −2.36) and 65.0 cm (<P5, height for age *Z* score −2.19), respectively. His serum total protein and albumin were 29.2 and 18.62 g/L, respectively. Detailed information regarding the laboratory tests is presented in Table 1. Another abdominal CT scan was conducted at our hospital and the result revealed that the patient still had intestinal malrotation (Figure 1B). During the laparoscopic procedure, intestinal malrotation with an almost 360° midgut volvulus was detected (Figure 2). Extensive lymphatic dilatation was observed in the bowel and mesentery. It was also found that a part of the small intestine had adhered to the mesentery. The small bowel was ischemic without necrosis. The patient was treated with Ladd's procedure, adhesiolysis, and an appendectomy. We also conducted a family-based WES evaluation to exclude monogenic disorders, with negative results. Biopsies during esophagastroduodenscopy at our hospital showed mild intestinal lymphangiectasia.

There was no need for repeated infusion of albumin and immunoglobulin 2 months post-surgery. The symptoms of diarrhea were completely relieved 6 months after the operation. The last follow-up was conducted 23 months post-operation. He did not have symptoms of chronic diarrhea or hypoalbuminemia. Although his nutritional status was improved, he still suffered from malnutrition. His weight and height were 12.0 kg (P10–P25, weight for age *Z* score −1) and 91.0 cm (P10–P25, height for age *Z* score −0.72), respectively.

## Discussion

Intestinal malrotation is often diagnosed in infancy and occurs at a rate of 1 in 2,500 live births; however, as an anatomic entity, it is much more common than this, occurring in 0.2%–1% of the normal population (3). Intestinal malrotation in newborns is usually diagnosed after signs of intestinal obstruction. However, in patients with atypical symptoms, the diagnosis may go unrecognized (2). In some patients, delayed diagnosis may lead to devastating consequences (4), but there is still no means of predicting which patients will progress to midgut volvulus or bowel ischemia.

Chronic midgut volvulus is less common than acute volvulus, and its symptoms may be less prominent. Chronic or recurrent volvulus can lead to mesenteric vascular and lymphatic obstructions, resulting in bowel edema and loss of protein-rich chyle into the bowel lumen, causing chronic diarrhea, hypoproteinemia, and failure to thrive. Malabsorption-like syndromes due to chronic midgut volvulus are rare. The diagnosis is often delayed because of subacute and atypical chronic symptoms. Although there have been reports of chronic diarrhea caused by intestinal malrotation as early as the 1980s (5,6), due to rare cases, clinicians may not often be aware that intestinal malrotation is the potential source of chronic diarrhea. In some cases, intestinal malrotation was detected, but it was overlooked by clinicians because of the atypical symptoms. Four Turkish children who primarily presented with long-standing diarrhea, hypoproteinemia, and failure to thrive secondary to intestinal malrotation were reported in 2004 (7). The average duration of symptoms was 35 months, and the longest delay in diagnosis was 6 years. In 2012, an infant with chronic diarrhea and PLE due to intestinal malrotation was reported (8). This patient presented with bilious vomiting in the neonatal period and underwent surgery immediately for jejunal duplication; however, intestinal malrotation was undetected or underappreciated, so the diagnosis was delayed by 8 months. This patient underwent extensive examinations until intestinal malrotation was incidentally detected on an abdominal MRI. In the two cases presented here, the disease onset was very early, both within the first week of life and they mainly presented with chronic diarrhea, protein-losing enteropathy, and failure to thrive. In one patient, an abdomen CT scan was conducted at the age of 3 months but intestinal malrotation was not detected. In the other patient, intestinal malrotation was detected 15 days postnatally but Ladd's procedure was not conducted. Due to unawareness that intestinal malrotation may be the cause of their chronic symptoms at the beginning, both patients underwent extensive examinations without a definitive etiology. This highlights that the malabsorption pattern is also one of the rare presentation modes of intestinal malrotation. However, to date, intestinal malrotation has not been included in diagnostic algorithms for the evaluation of chronic diarrhea or PLE (9–13).

Patients with intestinal malrotation often have other congenital malformations such as intestinal atresia, abdominal wall defects, a diaphragmatic hernia, Hirschsprung's disease, situs inversus, and cardiovascular defects. In some series, other concomitant congenital malformations are present in more than 50% of the cases (14). In some circumstances, intestinal malrotation may be overlooked or underappreciated by radiologists or clinicians, which may lead to a delayed diagnosis of the disease. In 2018, Herle and Halder (15) reported a case of a 40-year-old woman with a background of congenital abnormalities including Goldenhar and Klippel–Feil syndrome who presented with acute and chronic abdominal pain. Despite previous abdominal operations, namely, a cholecystectomy and an open duplex appendicectomy over 25 years ago, intestinal malrotation remained undiagnosed in this patient. In 2020, two infants with enteric duplication cysts and intestinal malrotation were reported (16). The first was a 4-month-old infant and the second was a 1.5-year-old boy. Each presented with recurrent attacks of bilious vomiting that proved to be due to acute midgut volvulus. In our report, one patient had mesenteric cystic lymphangioma. These indicate that intestinal malrotation and volvulus may be worth investigating in patients with known congenital disorders.

## Conclusion

Congenital diarrhea and enteropathies are a rare heterogeneous group of disorders. As a broad range of illnesses can present similarly in infants, establishing a definitive diagnosis of CODEs remains challenging. This report highlights the importance of considering intestinal malrotation in the differential diagnosis of obscure chronic diarrhea, especially in infants presenting with PLE or in combination with other congenital malformations.

## Data Availability

The original contributions presented in the study are included in the article/Supplementary Material, further inquiries can be directed to the corresponding author.
